# First Case of Diffuse Leishmaniasis Associated With *Leishmania panamensis*

**DOI:** 10.1093/ofid/ofy281

**Published:** 2018-11-23

**Authors:** Christian Olivo Freites, Nathan D Gundacker, Juan Miguel Pascale, Azael Saldaña, Rosendo Diaz-Suarez, Gabriela Jimenez, Nestor Sosa, Eyra García, Ana Jimenez, José A Suarez

**Affiliations:** 1 Instituto Conmemorativo Gorgas de Estudios de la Salud, Panama City, Panama; 2 Division of Infectious Diseases, Medical College of Wisconsin, Milwaukee; 3 Division of Infectious Diseases, Hospital Santo Tomás, Panama City, Panama

**Keywords:** clinical tropical medicine, diffuse cutaneous leishmaniasis, leishmania

## Abstract

*Leishmania panamensis* is the most common species of *Leishmania* in Panama, and it is known to cause cutaneous leishmaniasis, disseminated cutaneous leishmaniasis, and mucocutaneous leishmaniasis; however, it not associated with diffuse cutaneous disease. In this study, we report the first case of diffuse cutaneous leishmaniasis caused by *L panamensis*.

Cutaneous leishmaniasis (CL) is a sandfly-borne protozoan trypanosomatid endemic to tropical and subtropical countries around the world. Approximately 1.2 million cases of CL occur every year [1]. Cutaneous leishmaniasis is caused by an obligate intracellular protozoan trypanosomatid parasite. American CL (also called American tegumentary leishmaniasis) can present in multiple forms including the following: localized CL, disseminated CL (DL), diffuse CL (DCL), and mucocutaneous leishmaniasis (MCL). Cutaneous leishmaniasis is classically associated with a few open ulcers with raised borders at the site of a sandfly bite. Disseminated CL is characterized by a numerous lesions throughout the body, a low parasite load, and an overactive cell-mediated immune response resulting in tissue destruction. Mucocutaneous leishmaniasis is similar to DL but also causes destruction of mucosal surfaces and can occur years after the initial lesion. Diffuse CL is typically characterized as a nodular disease with an inappropriate humoral response that results in high parasite loads. Diffuse CL is difficult to treat, and many patients require multiple courses of antiparasitic therapy.

In the Republic of Panama, cases of CL, DL, MCL have been identified in our clinical practice. The incidence of CL in Panama is 90/100000 person-years [2]. *Leishmania panamensis* is the most common etiological agent of CL in Panama, causing localized CL [3], as well as DL [4] and MCL [5]. Diffuse CL is a rare type of CL, comprising only 0.1% of CL in endemic countries. Diffuse CL in the Americas has been previously associated with *Leishmania amazonensis*, *Leishmania Mexicana*, and *Leishmania piffanoi*, but not *L panamensis* [6–8]. Only 1 other case of DCL associated with *L panamensis* has been reported in Northwest Colombia in a patient with multiple nodular lesions; however, the speciation was questionable [9].

## CASE

**Figure 1.**
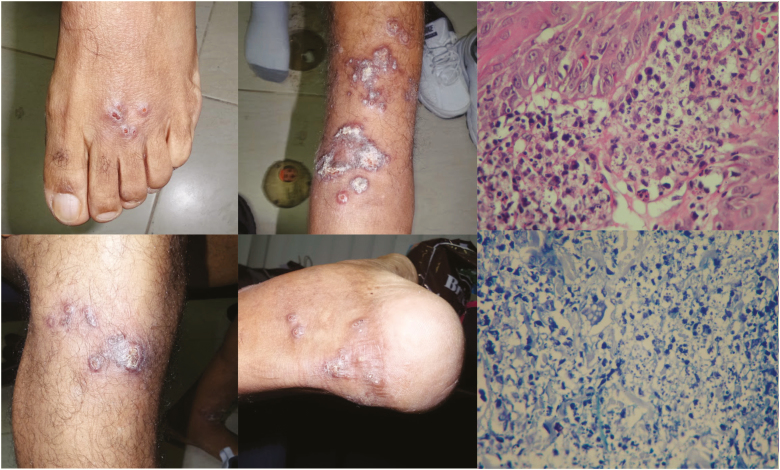
Diffuse cutaneous leishmaniasis lesions are nodular in nature with minimal open ulcerations. Mixed photomicrograph from top to bottom: intense inflammatory lympho-histiocytic reaction and high-power hematoxylin and eosin and Giemsa stains that show abundant intracellular amastigotes.

The patient is a 64-year-old Panamanian male physician, who is a resident in the Darien Province (a rural area endemic for CL), presented with multiple pleomorphic cutaneous lesions on his lower extremities for over 1 month. The patient states that approximately 2 months ago he went to a social event in Cerro Azul (mountainous area in the Panama Province, also an endemic area for leishmaniasis) where he received multiple bug bites. The patient first noticed a small hyperpigmented nodule on his thigh, which rapidly progressed to multiple lesions on both legs. The lesions were painless and nonpruritic. On presentation, he had 11 total lesions, spread over both lower extremities, more prominent in the legs and ankles. Two were located on the right posterior thigh, 2 on the right lower leg, 6 on the left ankle, and 1 on the left dorsal foot (Figure 1). Most of the lesions were nodular hyperpigmented lesions, whereas others were erythematous plaques. Some of these plaques had small areas of ulceration. No purulent secretions were seen. The patient’s initial work up showed the following: complete blood count, comprehensive metabolic panel, and erythrocyte sedimentation rate within normal limits. Venereal Disease Research Laboratory test, enzyme-linked immunosorbent assay, and Western blot for human immunodeficiency virus were negative. Montenegro and protein-purified derivate (PPD) skin tests were negative.

Biopsies were taken and sent for histopathology and polymerase chain reaction (PCR). Histopathology of the plaques and nodules revealed an intense chronic inflammatory reaction, epidermic ulceration with hyperkeratosis in the borders, a predominance of histiocytes, and mononuclear cells with numerous intracellular amastigotes in phagocytic vacuoles. Deoxyribonucleic acid (DNA) extraction of the biopsy was performed using the QIAGEN QIAmp DNA Blood Mini Kit according to manufacturer’s instructions (QIAGEN, Valencia, CA). The DNA extracted was amplified using oligonucleotide primers B1 and LV, which amplify the entire minicircle that specifically amplify the entire 750-base pair (bp) minicircle of *Leishmania Viannia* species. *Leishmania panamensis* was identified by a PCR analysis using oligonucleotides F25 and R1310, which amplify a 1286-bp product from the repeated gene heat shock protein 70 from the biopsy [10, 11]. *Leishmania Viannia panamensis*, *Leishmania Viannia brasiliensis*, and *Leishmania Viannia guyanensis* reference strains were used in this study as controls.

An ear/nose/throat evaluation including an endoscopy and computed tomography of head-neck were negative for mucosal disease. The patient was started on 20 mg/kg meglumine antimoniate (Glucantime; Sanofi Aventis, Suzano, Brazil) per day given intravenously for 20 days (this is the dose recommended by the ministry of health in Panama) with initial resolution of his symptoms. His disease recurred twice (all treated with the above-mentioned drug regimen) over a 2-year period. Recurrence was defined clinically by appearance of new lesions and reappearance or growth of the initial lesions. After 2 cycles of meglumine antimoniate without significant response, he was eventually treated with amphotericin B deoxycholate (total dose of 1.5 grams) with complete resolution of his lesions.

## DISCUSSION

This is the first case of *L panamensis* producing diffuse CL. Convit et al [7] first characterized this type of disease with the following criteria (Table 1): a localized lesion which disseminates to involve multiple sites on the skin with papular, nodular and plaque-like lesions that seldom ulcerate; absence of visceral lesions; specific anergy to leishmanin skin test antigen; in the skin biopsies, lesions that show large numbers of vacuolated macrophages with amastigotes; and a poor response to conventional therapy. Convit et al [7] also explained that this entity is most likely caused due to an unknown defect in the immune response against the parasite. Diffuse CL is characterized by an antibody-dependent immune response and the presence of multiple intracellular amastigotes. However, antibody-mediated immunity is ineffective against *Leishmania* amastigotes. In addition, patients typically have negative Montenegro and PPD skin tests. Patients with DCL show inhibition of natural killer cells and low levels of interferon (IFN)-γ and tumor necrosis factor-α [12]. These cytokines assist in granuloma formation and are essential to mount an effective cell-mediated response against the intracellular amastigotes. In addition, CD8 cells of patients with diffuse disease show characteristics of “exhaustion”, which include low cytotoxicity, low antigen-specific proliferation, and low IFN-γ production when compared with those with localized disease [13].

**Table 1.** Characteristics of Diffuse Cutaneous Leishmaniasis and Disseminated/Mucocutaneous Leishmaniasis

Characteristics	Diffuse Cutaneous Leishmaniasis	Disseminated/Mucocutaneous Leishmaniasis
Main agents	New World: - *Leishmania amazonensis* - *Leishmania mexicana* - *Leishmania piffanoi* - *Leishmania panamensis*	New World: *Viannia* species - *Leishmania braziliensis* - *Leishmania guyanensis* - *L panamensis*
Immune response	Th-2	Th-1
Lesional parasites	Abundant on pathology	Rare on pathology
Montenegro’s test	Negative/poor	Reactive
Plaque formation	Frequent	Rare
Response to drug therapy	Resistant/poor, relapse	Good
Infection/clinical course	Chronic, 20+ years	Acute, mucocutaneous leishmaniasis can occur years after primary lesion
Affected ages and sex	All ages and sexes	Young adults, male
Mucosal lesions	No	Yes

Treatment of patients with DCL is difficult because relapses are common. Many approaches have been tried with moderate success, including immunotherapy using *Leishmania* antigens and Bacillus Calmette-Guérin or in combination with antimoniate meglumine. Both antigen combinations appeared to be useful in Venezuelan patients with early lesions of DCL, but clinical improvement only occurred inpatients with chronic infection [14].

## CONCLUSIONS

The clinical immune response to leishmaniasis is an area of much-needed research. It is unknown which patients will develop disseminated, mucocutaneous, or diffuse disease. This case highlights that a species-specific response in leishmaniasis is not as predictive as previously thought. Although *L panamensis* is more likely to cause LCL and DL, a wide range of illnesses can likely occur including diffuse disease.
